# Insecticide-Treated Plastic Sheeting for Emergency Malaria Prevention and Shelter among Displaced Populations: An Observational Cohort Study in a Refugee Setting in Sierra Leone

**DOI:** 10.4269/ajtmh.2012.11-0744

**Published:** 2012-08-01

**Authors:** Matthew Burns, Mark Rowland, Raphael N'Guessan, Ilona Carneiro, Arlyne Beeche, Stefani Sesler Ruiz, Sarian Kamara, Willem Takken, Pierre Carnevale, Richard Allan

**Affiliations:** Wageningen University, Wageningen, The Netherlands; London School of Hygiene and Tropical Medicine, London, United Kingdom; International Development Research Centre, Ottawa, Ontario, Canada; The MENTOR Initiative, Crawley, United Kingdom; National Malaria Control Programme, Freetown, Sierra Leone; Institut de Recherche pour le Developpement (IRD), Montpellier, France

## Abstract

A double-blind phase III malaria prevention trial was conducted in two refugee camps using pre-manufactured insecticide-treated plastic sheeting (ITPS) or untreated polyethylene sheeting (UPS) randomly deployed to defined sectors of each camp. In Largo camp the ITPS or UPS was attached to inner walls and ceilings of shelters, whereas in Tobanda the ITPS or UPS was used to line only the ceiling and roof. In Largo the *Plasmodium falciparum* incidence rate in children up to 3 years of age who were cleared of parasites and monitored for 8 months was 163/100 person-years under UPS and 63 under ITPS (adjusted odds ratio [AOR] = 0.40, 95% confidence interval [CI] = 0.33–0.47). In Tobanda incidence was 157/100 person-years under UPS and 134 under ITPS (AOR = 0.85, 95% CI = 0.75–0.95). Protective efficacy was 61% under fully lined ITPS and 15% under roof lined ITPS. Anemia rates improved under ITPS in both camps. This novel tool proved to be a convenient, safe, and long-lasting method of malaria control when used as a full shelter lining in an emergency setting.

## Introduction

It has been estimated that up to one in three malaria-related deaths occurs in countries affected by conflict or natural disaster.^1^ Refugee and internally displaced populations are highly vulnerable to the effects of malaria, especially if migration occurs from areas of low to high transmission and the population is non-immune.^2^ The two principal methods of malaria vector control are indoor residual spraying (IRS) and insecticide-treated nets (ITNs), and both work well in endemic regions of Africa and South Asia when the infrastructure for timely supply or campaign planning is well established.^3^–^5^ During humanitarian crises, the feasibility of such tools is a major concern, given the demands placed on overstretched delivery agencies, operational constraints, the breakdown of social and public health networks, and the types of refugee shelter available. Times of crisis require fit-for-purpose, ready-to-use, readily stockpiled preventive tools that place no extra demands on hard pressed emergency services.^6^,^7^ During the last decade public and private sector organizations, under the leadership of the Roll Back Malaria (RBM) Partnership, have recognized the need to work together to bring complementary expertise to the task of identifying and developing vector control tools appropriate to humanitarian crises.^7^–^9^ Insecticide-treated polyethylene sheeting (ITPS), is one such tool emerging from this process and is being produced commercially.^10^ The ITPS is based on the standard polyethylene sheeting that is issued routinely as temporary shelter for people affected by emergencies. During manufacture the pyrethroid insecticide, deltamethrin, is extruded with the polyethylene into three-ply laminated sheets, comprising an inner low-density laminate and two, outer high-density laminates. The insecticide release characteristics enable the deltamethrin to diffuse slowly to the outer surfaces and to become available for pick-up by any insect that lands on the surface. Consequently, ITPS has a dual purpose: to provide shelter but with vector-control potential. Deployment and erection of ITPS is done in the same way as standard tarpaulin shelters. Until now, evaluation of ITPS has been limited to small-scale entomological testing in scientifically controlled environments “entomological platforms” in Asian^11^ and “experimental huts” in rural African settings.^12^,^13^ Before any novel control tool can go forward for recommendation by the World Health Organization (WHO), or be used routinely in humanitarian crises, clear demonstration of impact on malaria morbidity in emergency refugee settings is essential. A phase III field evaluation was therefore conducted to evaluate the impact of ITPS on malaria incidence in young children in an area of intense transmission. Secondary outcomes were associated with the impact of ITPS on anemia and adverse events (user safety). A unique feature of this trial was its setting—a true emergency—in two newly built refugee camps for Liberian refugees displaced to Sierra Leone. The findings offer insight into the effectiveness of ITPS when used in a scenario for which it was purposefully designed.

## Materials And Methods

### Study area and population.

**Figure 1.**
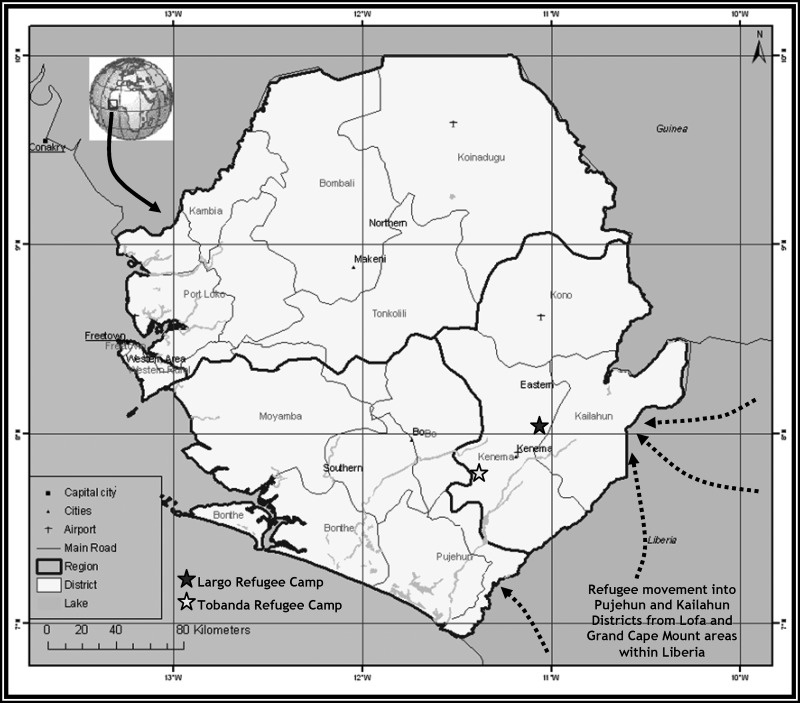
Map of Sierra Leone indicating evaluation sites and entry points of refugees from Liberia (dashed arrows).

The study was conducted in Largo (W 11.106; N 8.045) and Tobanda (W 11·364; N 7.797) refugee camps situated in southeast Sierra Leone, West Africa (Figure 1). The two camps were 70 km apart. The study area and malaria epidemiology have been described elsewhere.^14^,^15^ Malaria transmission is perennial.^16^ Mosquito fauna and population density are heterogeneous across the region.^15^,^17^,^18^ The most common causes of death in the local population are malaria and malnutrition, with a 61% reported prevalence of *Plasmodium falciparum* in children < 7 years of age in this area of Sierra Leone.^19^ This was similar to the prevalence reported in a similar age group in Liberia^20^ that indicates the refugees seeking asylum likely arrived from areas of similar malaria transmission. The principal vectors in this area of Sierra Leone were *Anopheles gambiae* s.l. and *Anopheles funestus* s.l.^18^ With no published evidence of insecticide resistance, the local vectors were considered to be fully susceptible to pyrethroid insecticides.

The United Nations High Commission for Refugees (UNHCR) negotiated the establishment of the refugee camps and provided basic shelter. Both camps were established to accommodate ∼8,000 refugees each. The camps were populated solely by Liberian refugees fleeing conflict in bordering provinces. The populations were represented by various ethnic groups from Lofa and Grand Cape Mount counties of Liberia. It was anticipated that refugees were likely to remain for at least 1–2 years, which was sufficient time to complete the study.

### Study design.

Taking into account the anticipated lifespan of the camps, the 2-year funding commitment from the Humanitarian Aid department of the European Commission (ECHO), and the time required for construction, refugee sensitization, and baseline monitoring, we elected to run a prospective cohort study of 8 months duration in which *P. falciparum* malaria incidence was monitored in children 4–36 months of age, the age group most at risk from malaria.^16^,^19^

**Figure 2.**
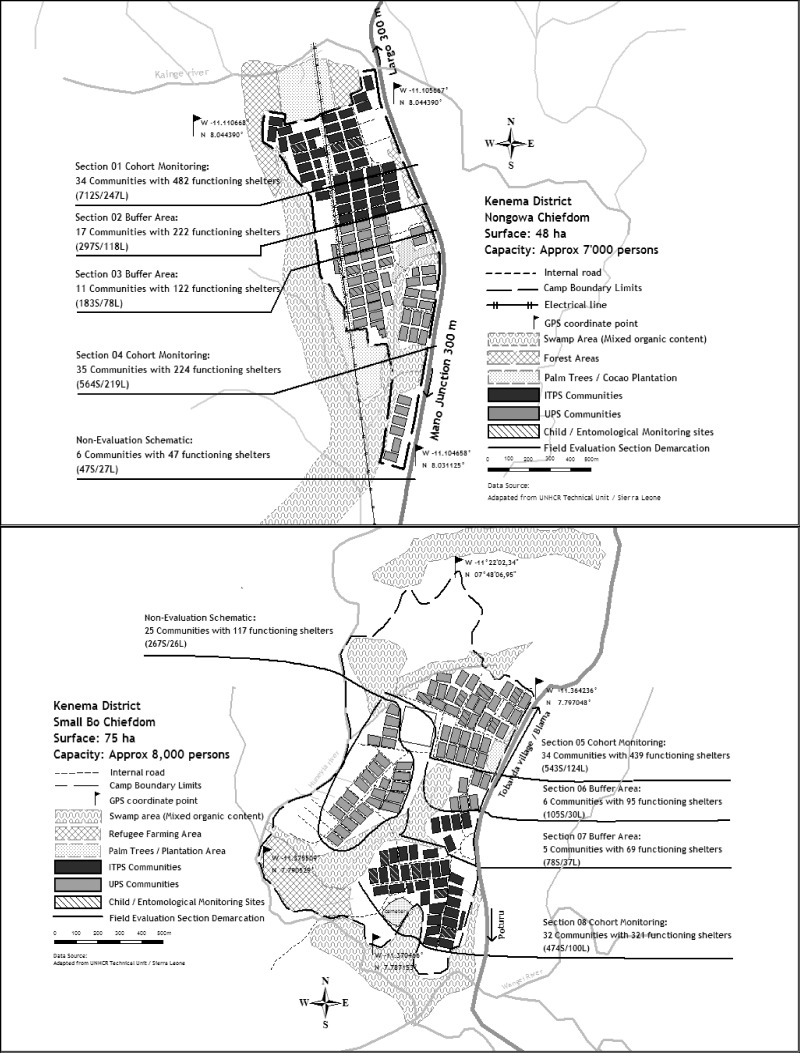
Largo (plots fully lined with polyethylene sheeting) and Tobanda (plot roof only covering with polyethylene sheeting). Refugee camps with intervention, control, and respective buffer section demarcation. Section information in parentheses refers to the maximum number of shelters (S) and latrines (L) that were planned for under initial camp design planning.

Each refugee camp was divided into four sections in which intervention (ITPS) and control untreated polyethylene sheeting (UPS) arms were randomly allocated to the outer sectors of each camp (Figure 2). The size of these outer sectors was based on reaching the appropriate buffer zone length of ∼0.5 km in length. The buffer zone consisted of two inner sections receiving ITPS or UPS as per their respective, adjacent, outer sectors, but without the epidemiological monitoring. The buffer zones helped to isolate the intervention from control sectors and served as reservoirs to absorb any repellent effect the ITPS might have on mosquito populations, which might otherwise inflate the real intervention impact.^21^–^23^ Each sector consisted of between 32 and 36 communities that on average contained 16 refugee family plots (shelter and verandah/garden) and 12 children 4–36 months of age.

Refugees were allocated individual family plots and provided with shelter kits by UNHCR. Shelter kits provided refugee families with the appropriate components to construct a wooden framed structure of 24 m^2^ (4 × 6 m) in which walls were rough casted from earth and a roof with eaves was covered with thatch. The shelter kits provided in both camps and within each sector were identical. The quantity of polyethylene sheeting distributed to families differed between the two camps. In the Largo camp all interior walls and ceilings were lined with either ITPS or UPS (full coverage). This design simulated the acute phase of a humanitarian crisis in which rudimentary shelters are constructed from polyethylene sheeting. The ITPS was attached to the roofing struts to create a plastic ceiling that met the tops of the four walls. The open eaves of the hut were set above the plastic ceiling layer thereby restricting mosquito entry into the living space via the eaves. By contrast, in the Tobanda camp, only enough ITPS or UPS was given out to form a roof and ceiling that was attached to the roofing mainframe. The ceiling-only coverage in Tobanda simulated usage of polyethylene sheeting in more established camps during the transition from acute to chronic phase emergency when families construct mud walled huts. The ITPS was identical to UPS in all respects apart from the pre-treatment with deltamethrin. Installation teams and refugee groups were blinded as to which polyethylene sheeting was treated or untreated. Both ITPS and UPS were produced by Vestergaard Frandsen, Denmark, under strict quality control that ensured batch separation between ITPS and UPS from manufacture to field delivery. Community latrines in the camps were also covered with UPS/ITPS in accordance with the respective sheeting type given to shelter coverage in each section of the camp. The time interval by which the majority of family plots had completed construction, and the earliest time in which monitoring of study cohorts could commence was 5–9 months in Largo and 3–5 months in Tobanda. Throughout the trial any UPS/ITPS that became storm damaged was replaced.

A tiered sensitization program ran concurrently with camp construction and the distribution of polyethylene sheeting. The purpose of the trial was explained to refugee leaders through camp meetings. Here, the reason for the randomized allocation of sheeting type was explained further and consensus to proceed with the trial was sought. Later tiers went into more detail about informed consent, the risks and benefits of participation, responsible use of polyethylene sheeting, and possible adverse events.

### Epidemiological monitoring.

**Figure 3.**
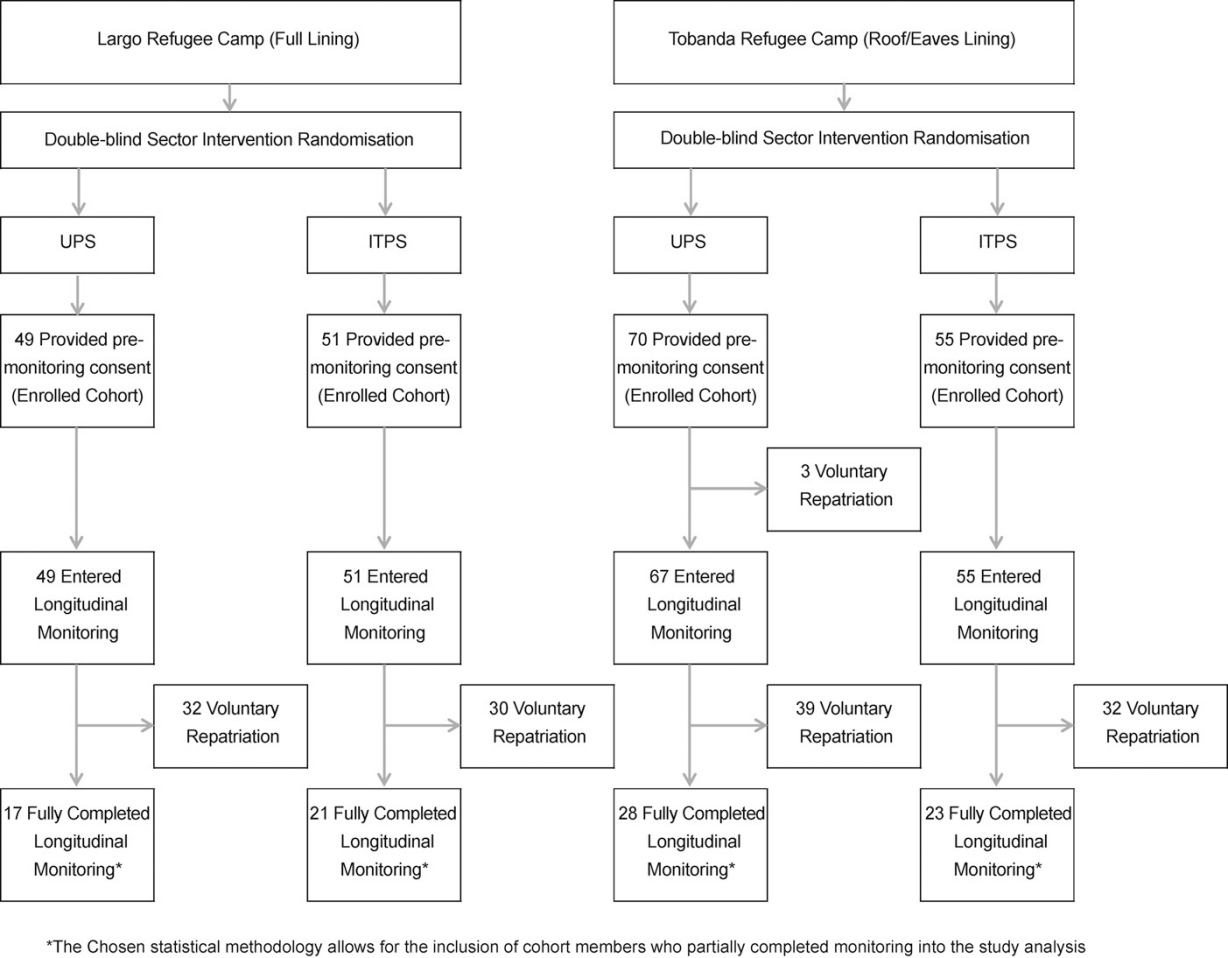
Study profile of child cohort (4–36 months) entered into longitudinal monitoring.

The primary outcome was the malaria incidence rate between children in each study arm. Children 4–36 months of age, taken equally from control and intervention arms were monitored as per the study profile (Figure 3). With an estimated incidence rate of 3–4 clinical episodes of malaria per child-year in the control cohorts,^24^ the sample size, taking into account intra-cluster correlation between children from the same communities was estimated to detect a 50% difference in incidence rate between intervention and control cohorts with 95% confidence and 80% power.

A baseline cross-sectional prevalence survey was conducted using Visitect (Omega Diagnostics, Scotland, United Kingdom) *P. falciparum* HRP-2 antigen detecting rapid diagnostic tests (RDTs), on a random sample of the whole population in ITPS and UPS arms in each camp in November 2003. This determined whether gradual exposure to ITPS during camp establishment and construction had affected parasite prevalence since arrival. Longitudinal monitoring of the child cohort commenced in December 2003 in both camps and terminated in July 2004.

Seven refugee communities in “full coverage” Largo and 12 in “ceiling/roof coverage” Tobanda were randomly selected for epidemiological monitoring. Location of the communities was stratified on the basis of distance from surrounding water-bodies (potential mosquito breeding sites) and the positioning of ITPS/UPS covered latrines (Figure 2).

In December 2003, all children 4–36 months of age within the clusters received directly observed 3-day combination therapy, with a daily dose of amodiaquine (10 mg/kg) and artesunate (4 mg/kg) from Co-treatment Blister Packs (Sanofi-Aventis, Gentilly Cedex, France) to provide clearance of any malaria parasites.^25^ Children were observed for 30 min post treatment in case of vomiting.

Inclusion criteria for the recruited children were residence in the camps, 4–36 months of age, and guardian informed consent. Children who had a serious illness other than malaria or who had experienced adverse reactions to amodiaquine or artesunate on a previous occasion were excluded from monitoring. Families were excluded if they anticipated moving out of the camp during the upcoming 12 months.

Between December 2003 and July 2004, daily monitoring of children in both camps was conducted from health screening points staffed by nurses and laboratory technicians who rotated between each screening point over the course of the study. Any child presenting with fever or reported fever in the last 24 hours was administered a clinical questionnaire based on the Integrated Management of Childhood Illness (IMCI), after which a RDT (as used in the baseline cross-sectional prevalence survey) was taken to confirm malaria positivity. Unwell children were referred to the camp health facility for further examination to identify any other potential causes of illness prior to treatment. If a child became ill outside of normal screening hours, they could attend the health facility and study nurses were notified the following day.

A malaria episode was defined as an individual with fever (≥ 37.5°C) or reported history of fever plus patent parasitemia confirmed by RDT. In addition to RDTs, a reference slide (thick and thin blood smears) were collected for confirmation of parasitemia. Unfortunately some of these deteriorated and were unreadable. Confirmation of malaria was therefore based on the RDT results.

In the study cohort hemoglobin (Hb) levels were monitored at 3 monthly intervals using a HemoCue photometer (HemoCue^®^, Ängelholm, Sweden) that was calibrated daily when used.

Symptoms or conditions considered to be potential adverse events related to ITPS included dizziness, inflamed/watery eyes, mucosal irritation, muscle cramps/tremors, nausea, runny nose, skin burning, skin itching, skin paraesthesia, skin rash, skin redness, sneezing, and tachycardia (pulse rate > 150). These symptoms or conditions were classified as potential adverse related events whether recorded on the IMCI assessment form or recorded directly as an adverse related event. This prevented the loss of any adverse events that were recorded as another child associated condition. A symptom listed repeatedly within a 7-day period for each child was considered to be the same adverse event as was any child having more than one of the symptoms present on a single day.

### Statistical analysis.

The baseline prevalence of malaria between control and intervention arms in each refugee camp was compared using a random effects multivariable logistic regression, adjusting for the potential intra-cluster correlation at the community level.

Malaria incidence rate was estimated as the total number of malaria episodes per person year over the course of the trial. This was analyzed using a random effects multivariable Poisson regression model to account for intra-cluster correlation among individuals from the same intervention clusters. The analysis was per protocol; any child who repatriated or became lost to follow-up was only included up to the date of loss or repatriation. The incidence rate ratios (IRR) between ITPS and UPS arms were estimated after adjusting for age and gender. Protective efficacy (PE) was calculated as (1 – IRR) × 100.

The time to first detected infection (symptomatic or asymptomatic) for children in UPS and ITPS arms was estimated using Kaplan-Meier survival analysis and log rank tests.

The effect of the intervention on anemia (Hb levels) was calculated by running a random effects linear regression, adjusting for repeated observations on the same children.

Differences in potential adverse events between treatment groups were analyzed using Pearson's χ^2^ test with Phi symmetric measure being used to measure the strength of association.

All analysis was performed using STATA 9 (StataCorp, College Station, TX).

### Ethical clearance and consent.

Approval for the study was obtained from the Ethics Committee for Research of the Sierra Leone Ministry of Health and Sanitation. The UNHCR Field Office granted permission for the study to be conducted in Largo and Tobanda refugee camps. The trial is registered on clincaltrials.gov with Identifier: NCT01456858. Refugees were fully informed about the trial and the risks/benefits before giving consent. Granting of informed consent was a two stage process. Heads of families gave informed consent to receiving at random ITPS or UPS. Heads of families who had children 4–36 months of age gave informed consent to longitudinal clinical and parasitological monitoring. Participation was voluntary. Refugee names were not recorded and locations were marked by only unique identification codes. Verbal information was given in English (the official language) and in the refugees' local language.

## Results

### Parasite prevalence.

A point prevalence survey was conducted before the cohort study on ∼11% of the population residing in Largo (*N* = 827) and Tobanda (*N* = 839). Although more females than males were sampled (61% female in Largo and 64% in Tobanda) the proportions were consistent in control and intervention arms (data not shown). Parasite prevalence was 54% (446 of 831) for Largo and 45% (355 of 779) for Tobanda. Having lived 5–9 months under polyethylene sheeting before the prevalence survey, the population in Largo under ITPS showed a decrease of 9.3% (95% confidence interval [CI]: 2.5%, 16.0%) in prevalence of infections compared with those living under UPS (Table 1 ). In Tobanda camp where exposure to polyethylene sheeting started 3–5 months before the prevalence survey, no significant difference in prevalence (2.6%, 95% CI = –4.4%, 9.5%) was observed between control and intervention arms either before or after adjusting for co-variables (Table 1). Prevalence of infection was highest in the 5–9 year age group.

### Malaria incidence.

Baseline characteristics of the study cohorts are shown in Table 2 . A total of 100 children from Largo and 122 from Tobanda were enrolled into the study. There was no significant difference in age distribution between UPS and ITPS groups in Largo (*P* = 0.338) or Tobanda (*P* = 0.9). Because of voluntary repatriation, 60% of the overall study cohort did not complete longitudinal monitoring (Figure 3). The loss to follow-up was similar between UPS and ITPS groups in both Largo (*P* = 0.252) and Tobanda (*P* = 0.49) and there was no significant difference in the age distribution of UPS and ITPS groups among those that completed the trial.

Table 3 summarizes the analysis of malaria incidence. The first 14 days of monitoring were omitted from analysis because this period is required to clear circulating falciparum HRP-2 antigen, which the RDT detects from peripheral blood. In Largo incidence was 163 and 63 per 100 child-years at risk in the control and intervention arms, respectively, and the adjusted incidence rate ratio (RR) was 0.40, giving a PE of 60% (*P* < 0.001). In Tobanda incidence was 157 and 134 per 100 child-years in the control and intervention arms, respectively, giving an adjusted incidence RR of 0.85 and a PE of 15% (*P* = 0.008). Adjustment for age and gender as potential confounding variables had a limited effect on the IRR point estimates.

**Figure 4.**
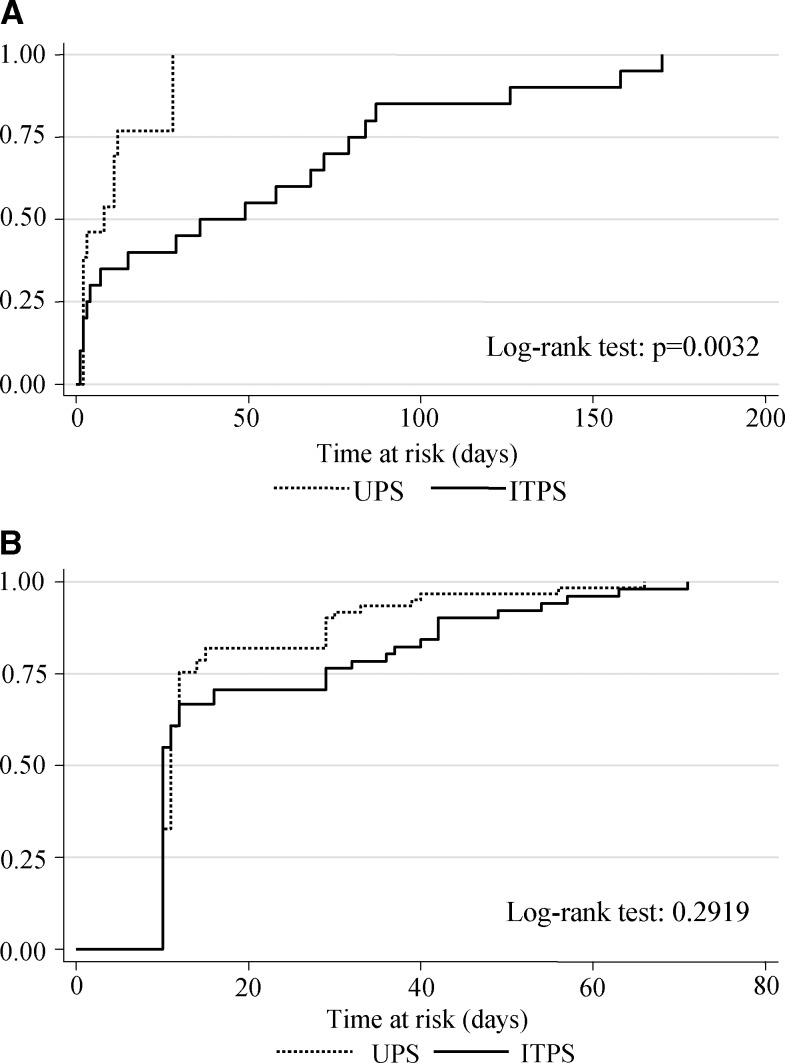
Kaplan-Meier failure analysis for time to first detected reinfection with *Plasmodium falciparum* among children 4 months to 3 years of age in (A) Largo refugee camp (ITPS, *N* = 51; UPS, *N* = 49) and (B) Tobanda refugee camp (ITPS, *N* = 55; UPS, *N* = 67).

Figure 4 shows Kaplan-Meier failure curves for the ITPS and UPS cohorts in each camp. In Largo camp, participants in the intervention arm took longer to become infected (log rank test; *P* = 0.0032) and the risk of malaria did not change over the course of the intervention. In Tobanda there was no significant difference between intervention and control arms (log rank test; *P* = 0.292).

### Anemia.

The mean Hb concentrations by survey and cohort groups are summarized in Table 4 . In Largo, the effect of the intervention was a significant increase in mean Hb of 0.6 g/dL (95% CI = 0.2, 0.9; *P* = 0.002), adjusted for survey and repeated sampling. Mean Hb was higher in the ITPS arm during the first survey, possibly indicating protection from ITPS during the camp construction period before the survey. There was a significant increase of 0.6 g/dL of mean Hb under ITPS at 3 months (95% CI = 0.4, 1.1; *P* < 0.001) and 0.7 g/dL at 6 months (95% CI = 0.3, 1.1; *P* < 0.001) compared with the baseline. The UPS also showed a similar level of increase in mean Hb over this interval (*P* < 0.001).

By contrast, there was no difference in mean Hb concentration at baseline between control and intervention groups in Tobanda (ceiling/roof only coverage camp). However over the 6 months of repeated surveys there was a significant increase in Hb of 0.7 g/dL (95% CI = 0.2, 1.1; *P* = 0.002) under ITPS compared with baseline, adjusted for survey and repeated sampling, but not in the UPS group.

### Adverse events.

Adverse related event occurrence among cohort groups is summarized in Table 5 . In both camps no significant differences between ITPS and UPS were observed from symptoms that could be linked either directly or indirectly to an adverse recorded event. All individual adverse related events were fully resolved before the end of the child monitoring period.

## Discussion And Conclusions

The trials showed that ITPS has the potential to be an effective tool for community control of malaria in emergencies depending on how the sheeting is used. Where the ITPS was used to cover walls and ceilings, as in Largo camp, the incidence of childhood malaria was reduced considerably. This method of deployment simulates newly erected refugee camps in acute phase emergencies in which polyethylene sheeting is used to form both the roof and walls of shelters. Although it remains unclear at which point during the 9-month establishment of Largo the ITPS started to provide protection, by the time longitudinal monitoring was able to start, the prevalence of malaria in the overall population was significantly reduced in the ITPS sectors relative to UPS sectors. Given the downward trend in malaria incidence from that point onward, it is reasonable to assume that the protection afforded by ITPS extended well beyond the formal 8-month period of longitudinal monitoring. It is conceivable that the difference in parasite prevalence between ITPS and UPS sectors recorded at the start of longitudinal monitoring reflected a difference between populations on admission. There was no other difference between the origin, recruitment, and characteristics of these two groups and we therefore consider it more likely the difference in prevalence was caused by an intervention effect during the construction phase that continued during the subsequent period of longitudinal monitoring. With the benefit of hindsight, prevalence of infection should have been recorded at arrival however this was not feasible when camps were in early development.

In the Tobanda refugee camp where the plastic sheeting was used only to line the ceilings and roof, no such difference in prevalence was observed between ITPS and UPS sectors. This mode of use simulated the redeployment of ITPS as roofing material in chronic emergencies when refugee families start to abandon their makeshift plastic shelters and construct homes from local materials. The contrast between Largo and Tobanda is most likely caused by differences in the way the ITPS was positioned within the dwellings, as the PE of ITPS correlates with the area of coverage within the rooms. When used to cover the inner walls and ceilings of shelters, ITPS offered 60% protection against transmission compared with only 15% protection in the camp where ITPS was used for only ceiling and roof lining. The full lining of walls and ceilings in Largo provided a greater surface area for mosquito vector contact. The ITPS works rather like IRS where impact comes from mosquitoes at rest on treated surfaces after a blood meal.^11^ Saturation coverage with ITPS in the intervention sector of Largo would provide few untreated surfaces upon which blood-fed mosquitoes can alight.

The observed impact of ITPS in Largo is consistent with the results of an entomological trial conducted in experimental huts in Burkina Faso, a trial in which the efficacy of ITPS was evaluated at different levels of wall and ceiling coverage.^12^ Experimental huts provide controlled conditions to observe the biting or blood feeding rates and mortality rates induced on mosquitoes by insecticide interventions. In the Burkina Faso trial the percentage of mosquitoes killed was dependent upon the room surface area covered with ITPS. The ITPS on all four walls and ceiling produced higher mortality (60%) of hut-entering mosquitoes than when ITPS was confined only to the ceiling (10% mortality). The malaria control trial in the Sierra Leone camps indicated that epidemiological impact in children also correlates with the proportion of insecticidal surface available.

This study confirmed the effectiveness of ITPS as a dual purpose tool for controlling malaria. Its value lies in combining shelter provision with disease prevention. This should simplify and accelerate delivery of appropriate aid in humanitarian crises and maximize uptake and coverage of protection amongst displaced communities. The ITPS provides levels of protection similar to other acclaimed techniques for malaria prevention, namely ITNs and IRS, which are far less amenable for implementation immediately after natural disaster or conflict. The PE of 60% found in Largo camp was similar to the PE shown by ITNs in Ivory Coast and Malawian child cohorts with a similar age profile.^26^,^27^ The loss of protection when the ITPS was used as roof-only lining indicates that when conditions evolve from acute to chronic emergency and refugee families start to construct more permanent homes, this would be an appropriate time to switch the control intervention from provision of ITPS to provision of long lasting insecticidal nets.^3^

Full coverage of ITPS reduced the prevalence of anemia. Hemoglobin levels increased during the monitoring period not only among the group under ITPS but also among those under UPS. Because changes in house design alone have been shown to reduce malaria,^28^,^29^ the health benefit gained in sleeping under UPS might be partly attributable to the simple improvements in shelter design (full interior lining), which might have reduced access to host-seeking mosquitoes. Increases in Hb also occurred in Tobanda camp. The improvements in Hb levels occurred during the time when the camp health infrastructure developed, and could also be caused by wider access to health care for treatment of malaria and non-malaria infections together with supplementary feeding and nutritional support to refugees.

When ITPS is distributed and used under operational conditions, it provides no further risk of adverse effects to beneficiaries than standard plastic sheeting. Provided that standardized field operating procedures and safety instructions are followed by distributors and end-users, ITPS would then be as safe to use as LLINs and IRS.

Our field evaluation was not truly cluster randomized because constraints on study design imposed by the size and structure of the two refugee camps. These limitations contrast with typical local rural villages that are small and dispersed and therefore more suited to cluster randomized designs. The many practical and logistical constraints in emergencies limit the available options. Smaller scale cluster randomization within the camp was considered. However, as is the case for IRS, the area under ITPS is required to be sufficiently large to induce a “mass killing effect” of the mosquito population that is necessary for malaria control.^11^ Alternative study designs such as a mosaic of smaller randomized UPS/ITPS clusters throughout the camp was not feasible, and any mosquito movement between the smaller sized UPS and ITPS clusters would obscure any differential effect of ITPS on the local mosquito population or on malaria transmission. In an ideal design the study's refugee camps would have been smaller and more separated to allow greater scope for cluster randomization.

Distribution of ITPS to provide high surface area coverage (walls and roofing) offers an effective and safe tool for malaria control in displaced populations. The ITPS supplied as only roofing treatments are not recommended on their own as any effect would be insufficient to control malaria. The use of ITPS is a viable option in acute phase emergencies. As the situation stabilizes and refugee population's switch from polyethylene shelters to construction of habitations from local materials, LLINs should be used for protection. Distribution of ITPS is easier to organize than IRS or LLINs at the outset of a humanitarian crisis and may provide efficiencies in program costs and speed of delivery compared with separate shelter and malaria control interventions. Consideration should be given to the routine supply of ITPS in emergencies in malaria-endemic areas.

## Figures and Tables

**Table 1:** Cross-sectional population survey of *Plasmodium falciparum* prevalence in samples of the refugee populations after the camps were fully established and before the longitudinal monitoring of the child cohorts*

Largo (full shelter coverage)		% Positive	(No. pos/total)	OR	95% CI	AOR	95% CI	*P*
Treatment	UPS	58.2	(248/426)	1		1		
	ITPS	48.9	(198/405)	0.68	(0.52, 0.90)	0.57	(0.41, 0.78)	0.001
Age group	< 5 y	73.8	(183/248)	1		1		
	5–9 y	86.1	(105/122)	2.21	(1.23, 3.98)	2.21	(1.23, 3.98)	0.008
	10–14 y	69.7	(46/66)	0.87	(0.47, 1.58)	0.87	(0.48, 1.60)	0.66
	> 14 y	28.1	(110/391)	0.13	(0.09, 0.19)	0.13	(0.09, 0.19)	0.0001
Gender	Male	59.3	(192/324)	1		1		
	Female	50.3	(254/505)	0.70	(0.53, 0.93)	1.01	(0.72, 1.40)	0.97
Tobanda (ceiling/roof coverage)		% positive	(No. pos/total)	OR	95% CI	AOR	95% CI	*P*
Treatment	UPS	44.4	(186/419)	1		1		
	ITPS	46.9	(169/360)	1.11	(0.84, 1.47)	1.09	(0.80, 1.48)	0.59
Age group	< 5 y	56.5	(135/239)	1		1		
	5–9 y	73.9	(105/142)	2.19	(1.39, 3.44)	2.27	(1.41, 3.67)	0.001
	10–14 y	59.4	(41/60)	1.13	(0.65, 1.94)	0.97	(0.55, 1.70)	0.92
	> 14 y	25.7	(100/389)	0.27	(0.19, 0.38)	0.24	(0.17, 0.35)	0.0001
Gender	Male	50.5	(151/229)	1		1		
	Female	42.5	(229/539)	0.73	(0.55, 0.96)	0.96	(0.69, 1.33)	0.81

* OR = odds ratio; CI = confidence interval; AOR = adjusted odds ratio; UPS = polyethylene sheeting; ITPS = insecticide-treated plastic sheeting.

**Table 2:** Cohort characteristics post-camp construction and before longitudinal monitoring*

	Largo		Tobanda	
	UPS	ITPS	UPS	ITPS
Study population size	49	51	67	55
Mean age in years (SE)	2.5 (0.11)	2.3 (0.13)	2.0 (0.13)	2.0 (0.14)
Gender: % male	53	57	49	45
Mean hemoglobin (CI)	9.3 (8.9, 9.6)	9.9 (9.6, 10.2)	9.3 (8.9, 9.7)	9.6 (9.2, 10.0)

* Pre-exposure to insecticide-treated plastic sheeting (ITPS) was 5–9 months during the establishment of Largo and 3–5 months during the establishment of Tobanda. Surface area coverage in Largo was maximized through lining the interior walls and ceilings but in Tobanda was limited to ceilings and roofs.

UPS = polyethylene sheeting.

**Table 3:** Incidence rates and ratios of *Plasmodium falciparum* infection among control and intervention groups in Largo and Tobanda camps, estimated from the random effects Poisson regression model*

	Number enrolled	Total child days at risk	Average number of days at risk	IR (95% CI)	IRR (95% CI)	% PE (95% CI)	Wald test *P* value	Adjusted IRR† (95% CI)	Adjusted % PE† (95% CI)	Wald test *P* value
Largo										
UPS	49	6801	139	163 (157–169)	1	–		1	–	
ITPS	51	8459	166	63 (52–76)	0.39 (0.36–0.41)	61 (59–63)	< 0.001	0.40 (0.33–0.47)	60 (53–67)	< 0.001
Tobanda										
UPS	67	9102	136	157 (147–167)	1	–		1	–	
ITPS	55	6661	121	133 (120–148)	0.85 (0.81–0.89)	15 (11–19)	0.008	0.85 (0.75–0.95)	15 (5–25)	0.008

* IR = incidence rate per 100 child-years at risk; IRR = incidence rate ratio; PE = protective efficacy; UPS = polyethylene sheeting; ITPS = insecticide-treated plastic sheeting; CI = confidence interval.

† Adjusted for age and sex.

**Table 4:** Mean (standard deviation) hemoglobin (Hb) levels in child cohorts during cross-sectional surveys after 0, 3, and 6 months of epidemiological monitoring*

	UPS		ITPS	
	*N*	Mean Hb (g/dl) [sd]	*N*	Mean Hb (g/dl) [sd]
Largo				
Survey 1 (baseline)	48	9.3^a^ [1.2]	51	9.9^a^ [1.1]
Survey 2 (3 months)	30	10.1^b^ [1.0]	34	10.5^b^ [1.2]
Survey 3 (6 months)	19	10.0^b^ [1.2]	21	10.6^b^ [1.2]
Tobanda				
Survey 1 (baseline)	59	9.3^a^ [1.4]	52	9.6^a^ [1.5]
Survey 2 (3 months)	36	9.1^a^ [1.1]	29	10.3^b^ [1.6]
Survey 3 (6 months)	33	9.3^a^ [1.1]	24	10.6^b^ [0.9]

* Values in the same column sharing a superscript do not differ significantly (at *P* < 0.05 level).

**Table 5:** Prospective adverse events (AE) recorded from child cohort longitudinal monitoring visit records between treatment arms*

		Average AE event per child (min-max)	AE events recorded (%)	Total monitoring visits	*P*	Phi effect
Largo	UPS	9.6 (0–28)	498^a^ (10)	4989	0.329	0.09
	ITPS	12.4 (2–24)	596^a^ (11)	5645		
Tobanda	UPS	8.7 (0–27)	579^a^ (9)	6118	0.699	0.04
	ITPS	8.6 (0–21)	459^a^ (10)	4740		

* Values in the same column sharing a superscript do not differ significantly (at *P* < 0.05 level).
